# Transcriptome Analysis of Androgenic Gland for Discovery of Novel Genes from the Oriental River Prawn, *Macrobrachium nipponense*, Using Illumina Hiseq 2000

**DOI:** 10.1371/journal.pone.0076840

**Published:** 2013-10-28

**Authors:** Shubo Jin, Hongtuo Fu, Qiao Zhou, Shengming Sun, Sufei Jiang, Yiwei Xiong, Yongsheng Gong, Hui Qiao, Wenyi Zhang

**Affiliations:** 1 Wuxi Fishery College, Nanjing Agricultural University, Wuxi, People Republic of China; 2 Key Laboratory of Freshwater Fisheries and Germplasm Resources Utilization, Ministry of Agriculture, Freshwater Fisheries Research Center, Chinese Academy of Fishery Sciences,Wuxi, People Republic of China; Macquarie University, Australia

## Abstract

**Background:**

The oriental river prawn, *Macrobrachium nipponense*, is an important aquaculture species in China, even in whole of Asia. The androgenic gland produces hormones that play crucial roles in sexual differentiation to maleness. This study is the first *de novo M. nipponense* transcriptome analysis using cDNA prepared from mRNA isolated from the androgenic gland. Illumina/Solexa was used for sequencing.

**Methodology and Principal Finding:**

The total volume of RNA sample was more than 5 ug. We generated 70,853,361 high quality reads after eliminating adapter sequences and filtering out low-quality reads. A total of 78,408 isosequences were obtained by clustering and assembly of the clean reads, producing 57,619 non-redundant transcripts with an average length of 1244.19 bp. In total 70,702 isosequences were matched to the Nr database, additional analyses were performed by GO (33,203), KEGG (17,868), and COG analyses (13,817), identifying the potential genes and their functions. A total of 47 sex-determination related gene families were identified from the *M. nipponense* androgenic gland transcriptome based on the functional annotation of non-redundant transcripts and comparisons with the published literature. Furthermore, a total of 40 candidate novel genes were found, that may contribute to sex-determination based on their extremely high expression levels in the androgenic compared to other sex glands,. Further, 437 SSRs and 65,535 high-confidence SNPs were identified in this EST dataset from which 14 EST-SSR markers have been isolated.

**Conclusion:**

Our study provides new sequence information for *M. nipponense*, which will be the basis for further genetic studies on decapods crustaceans. More importantly, this study dramatically improves understanding of sex-determination mechanisms, and advances sex-determination research in all crustacean species. The huge number of potential SSR and SNP markers isolated from the transcriptome may shed the lights on research in many fields, including the evolution and molecular ecology of *Macrobrachium* species.

## Introduction

The oriental river prawn, *Macrobrachium nipponense* (Crustacea; Decapoda; Palaemonidae), is an important commercial prawn species, that is widely distributed in freshwater and low-salinity estuarine regions in China and other Asian countries [1]–[7] with an aquaculture production of 205,010 tons annually for aquaculture only [8]. As known, within many other *Macrobrachium* species, the males grow faster and gain more weight at harvest time than females. Facing stiff market competition, *Macrobrachium* producers require improvement in fish production and performance traits to obtain more profit. Thus, culture of all-male populations would be necessary for economic purpose. Therefore, the long-term goals of the *M. nipponense* aquaculture industry include making genetic improvements and gaining a better understanding of sex-differentiation in this species. Some cDNA libraries and transcriptome-level datasets have been generated and serving as a basis for functional genomics approaches aimed at improving the aquaculture performance of this species [9]–[11]. However, little information on sex-determination and sex-differentiation related genes in the androgenic gland of *M. nipponense* have been reported, therefore, the mechanism of sex-determination remains unclear.

The androgenic gland is found in most crustacean species, it produces hormones that play crucial roles in sexual differentiation to maleness, including the development of the testes and male sexual characteristics [12]. Androgenic gland hormone (AGH) and its relative producers are recognized as important factors when the sex-differentiation and determination mechanisms are concerned, their functions have been widely studied in many crustaceans [13]–[15]. In *Macrobrachium rosenbergii*, the males undergoes sex reversal to the females after androgenic gland was ablated from males. The all-male population was generated when the “reversal females” were mating with normal male M. rosenbergii [16]–[18]. Considering the fact that ablation or implantation of the androgenic gland at a certain stage of development can result in sex reversal to male or female [12], [19]–[21], the expression pattern of androgenic gland hormone genes in crustaceans have received much attention in recent years [22]–[27]. Previous studies have focused on morphology and anatomy on the *M. nipponense* androgenic gland [28]. However, no information to date was reported on genetic underpinnings of the androgenic gland in *M. nipponense*. More novel genes, potentially involved in the sex-determination mechanism, are necessary to be identified from the androgenic gland. Such basic information is essential to better understand the molecular mechanisms involving in sex-determination/differentiation, in turn, it will benefit monosex crustacean aquaculture production.

The transcriptome is the total set of transcripts, mRNA, and non-coding RNA, in one or a population of cells during a specific developmental stage or in response to a particular physiological condition using high-throughput technology [29]. Transcriptome analysis provides the foundation for gene structure and function research and determines when genes are expressed and how they are regulated. The development of next-generation sequencing (NGS) technologies allows the acquisition of more sequence data per run at a substantially lower cost than in long-read technologies [30]. Compared with the microarray methods, NGS, developed by Illumina/Solexa, is able to generate over one billion bases of high-quality sequences per run at less than 1% of the cost of capillary-based methods, and is expected to dominate future analysis of eukaryotic transcriptomes [31]–[34]. Transcriptome analysis is now being widely applied to elucidate genetic factors conferring economically significant traits and/or phenotypes and to manage genetic diversity in cultured crustacean species [9]–[11], [35]–[38]. To date, there are only 81,411 expressed sequence tags (ESTs) from *M. nipponense* in the public databases, but the classical androgenic gland genes are largely absent from these gene sets [11].

In this study, we generate the first *M.* nipponense transcriptome using cDNA prepared from mRNA which was isolated from the androgenic gland. The EST sequences generated were assembled and annotated with putative functions where possible, and database mining was performed to identify sex-determination and differentiation related genes. A variety of markers potentially useful for genomic population studies including simple sequence repeats (SSRs) located within coding regions and single nucleotide polymorphisms (SNPs) detected amongst deep coverage sequence region reads are also reported.

## Results and Discussion

### Transcriptome Analysis

**Figure 1:**
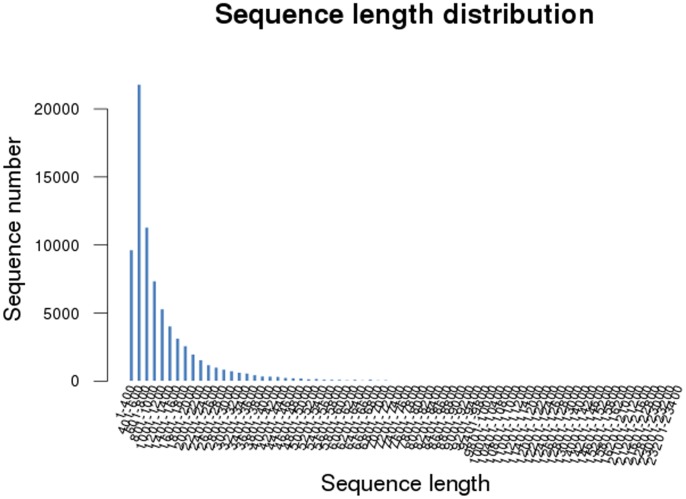
Contig length distribution of *M. nipponense* transcriptomic ESTs.

To the best of our knowledge, this is the first comprehensive study of the *M. nipponense* androgenic gland transcriptome sequenced by Illumina/Solexa. In the present study, the transcriptome profile was used to study the sex-determination and differentiation genes in the androgenic gland using next-generation sequencing technologies. Interestingly, the results revealed that there were genes highly expressed in the androgenic gland, which may be potentially involved in the regulatory mechanism of sex-determination in the oriental river prawn. The reads produced by the Illumina Hiseq2000 were used for clustering and *de novo* assembly. The reads yielded a total of approximately 357.8 million 200 bp paired-end raw reads with a total size of 7,229,376,990. After eliminating adapter sequences and filtering out the low-quality reads (the number of bases in each read was less than 25 bp) before *de novo* assembly by the SeqPreq program, Illumina Hiseq2000 sequencing yielded a total of 70,853,361 (**Table S1, Table S2**) high-quality transcriptome reads with a total size of 6,841,680,044 bp. *M. nipponense* is a non-model organism and has no reference genome sequence. Therefore, the raw data were assembled *de novo* using the Trinity program resulting in 78,408 contigs, ranging from 351 to 23,217 bp (**Table 1**). Most of the contigs (27.8%) were 401–600 bp in length, followed by 601–800 bp (14.42%), and 1–400 bp in length (12.3%) (**Figure 1**). These 78,408 isosequences yielded a total of 57,619 non-redundant transcripts with an average of 1,244.19 bp because of alternative splicing, thus, two or more isosequences may be matched to one transcript. In prawn, the earliest cDNA libraries were constructed in 2001, based on hemocytes and hepatopancreas from *Litopenaeus vannamei* and *L. setiferus*, in order to discovery immune genes and a total of approximately 2045 randomly selected clones were sequenced [39]. Three cDNA libraries (based on the material from the testes, ovaries, and milti-tissues) were constructed in previous studies on *M. nipponense* by Roche 454 GS FLX sequencing [9]–[11]. However, only limited numbers of genes were obtained from these three cDNA libraries. Compared with those studies, a great number of genes were generated in the current study, taking advantage of Illumina Hiseq2000 NGS which can sequence in higher throughput and provide more candidate genes. Besides, the average length of each isosequence is much longer than the previous studies after clustering and de novo assembly in current study, which can promote further studies on these isosequences, including RT- PCR and western-blot. The 57,619 non-redundant transcripts in this study provide a transcriptome database for future analyses of sex-determination and sex- differentiation related genes in androgenic gland tissue. Therefore, this transcriptome dataset accelerates the understanding toward the sex-determination mechanisms in *M. nipponense*, and other crustaceans.

**Table 1:** Summary of Illumina Hiseq2000 assembly and analysis of *M.nipponense* transcriptomic sequences.

	Number
Total genes	57619
Total isogenes	78408
Total residues	97554839
Average length	1244.19
Largest isogene	23217
Smallest isogene	351

### Gene Ontology Assignments and COG Analysis

To identify their putative functions, all of the isosequences were compared with the non-redundant protein database and nucleotide sequences in NCBI using Blastp and Blastx at an E-value of <10^−5^ in the priority order of the Kyoto Encyclopedia of Genes and Genomes (KEGG) database and Cluster of Orthologous Groups (COG) database.

A total of 70,702 non-redundant transcripts, which matched Nr, were annotated, while the other unannotated transcripts represent novel genes whose functions have not yet been identified. These unannotated transcripts may also play a vital role in the metabolism of *M. nipponense*; however, further research is required. A total of 5,365 out of 78,408 isosequences contain ORFs, with an average protein length of 719.3 bp and a mean nucleotide length of 2,157.9 bp. This implies that these 5,465 isosequences, which might be translated into amino acids, play an essential role in *M. nipponense* metabolism. Additional function analysis of these isosequences conducted using the COG, GO, and KEGG pathway databases are necessary.

**Figure 2:**
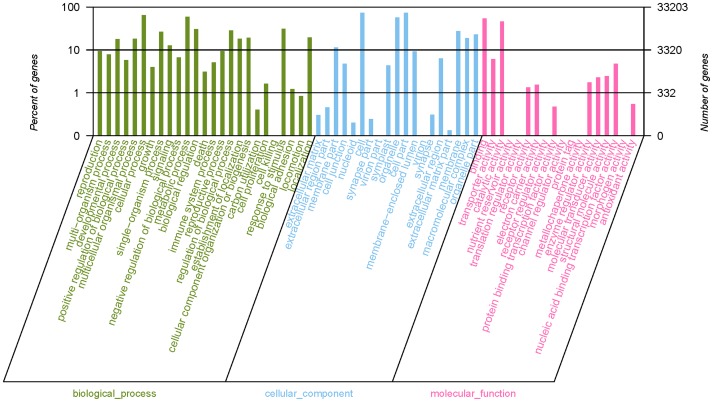
Gene ontology classification of non-redundant transcripts. By alignment to GO terms, 33203 isogenes were mainly divided into three categories with 62 functional groups: biological process (25 functional groups), cellular component (19 functional groups), and molecular function (18 functional groups). The left y-axis indicates the percentage of a specific category of genes existed in the main category, whereas the right y-axis indicates the number of a specific category of genes existed in main category.

Gene Ontology (GO) divides gene products into three categories (molecular function, cellular component and biological process), aimed to provide a structured and controlled vocabulary for describing gene products. GO terms were assigned to 33,203 *M. nipponense* contigs based on BLAST matches to proteins with known functions, including 40,399 sequences assigned to the molecular function category, 102,835 to the cellular component category, and 133,527 to the biological process component (**Table S3**). The matched contigs were comprised of 62 functional groups (**Figure 2**). Gene Ontology (GO) can provide a structured and controlled vocabulary for describing gene products in three categories: molecular function, cellular component, and biological process [40]. Analyses of the transcriptomes of other crustaceans have identified ESTs possessing similar arrays of potential metabolic functions [9]–[11], [35]–[38]. The total number of GO terms in this study was much larger than that of unique sequences, because many contigs can be assigned to more than one GO term. In the molecular function category, the number of contigs in each GO term ranged from 1 to 24,163. Cell, cell part, and cellular process had most abundant contigs (>20,000). However, there were also 9 functional groups in which the numbers of contigs were less than 10.

**Figure 3:**
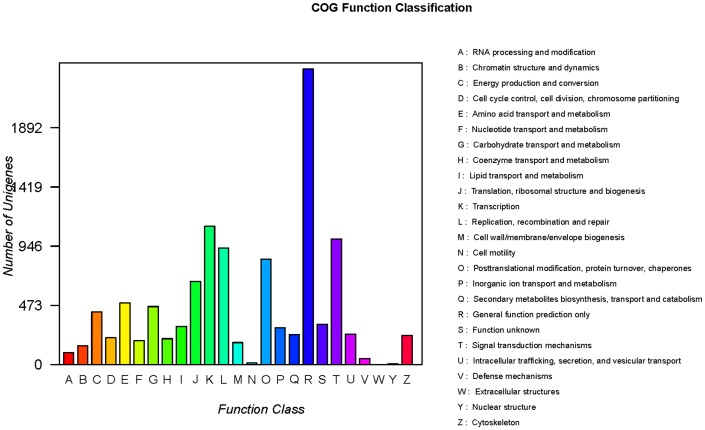
Cluster of orthologous groups (COG) classification of putative proteins. A total of 13817 putative proteins were classified functionally into 25 molecular families in the COG database.

The additional analysis revealed that 13,817 isosequences matched known genes in the COG database. Based on their predicted functions, these unigenes were classified into 25 functional categories (**Table S4**). Among these 25 functional categories, a cluster for General function prediction only represents the largest group with 4,162 unique sequences, followed by signal transduction mechanisms (2,258), posttranslational modification, protein turnover, chaperones (1,947), and transcription (1,785). Clusters for cell motility, extracellular structures, and nuclear structure represent the smallest groups, in which the numbers of sequences were <30 (**Figure 3**). Similar to the data in the GO category, the total number of COG sequences was >13,817 because several sequences were involved in more than one functional category.

The results obtained in this study showed that candidate novel genes with special functions can be easily identified according to the Gene Ontology assignment and COG analysis. Biological functions were mostly predicted to be involved in developmental process, growth and cell proliferation functional groups in “GO assignments” and the general functional prediction only and signal transduction mechanisms functional categories in “COG analysis”, which are more likely to contain the sex-determination and differentiation related genes in *M. nipponense*. The genes in these functional groups in current study were dramatically more abundant than those in previous studies in *M. nipponense* [9]–[11], providing more candidate selections for further analysis of sex-determination and differentiation mechanism in *M. nipponense*.

### KEGG Analysis

KEGG database can map the unique sequence into defined metabolic pathway. KEGG analysis was used to identify the potential candidate transcripts in biological pathways in the ladybird. A total of 17,868 isosequences matched the metabolic pathways in the KEGG Pathway database, mapped onto 317 predicted metabolic pathways, and grouped into amino acid metabolism, genetic information processing, cellular processes, and environmental information processing (**Table S5**). The numbers of unique sequences mapped to various pathways ranged from 1 to 3,500. The main metabolic pathways of unique sequences in the M. nipponense transcriptome are metabolic pathways, biosynthesis of secondary metabolites, microbial metabolism in diverse environments, spliceosome, RNA transport, protein processing in the endoplasmic reticulum, and purine metabolism, in which the numbers of unique sequences were more than 400. Identifying these pathways in the database was acceptable, considering the growth process habits of *M. nipponense*. KEGG analysis dramatically advances the researches on the relationship between different genes in the transcriptome of androgenic gland in depth. Some metabolic pathways, including spliceosome, and RNA degradation may promote the analysis of sex-determination and differentiation mechanism, because many sex-determination and differentiation related genes were existed in these pathways, such as Transformer-2, and the series of heat-shock protein family. Although not all of the major genes reported in the putative KEGG pathways were found in the current study, this information provides insight into the specific responses and functions involved in the molecular processes of *M. nipponense* metabolism and sex-determination.

### Research on Sex- related Genes

Sex determination is a fundamental and very important biological process involved in the development of sexual characteristics in organisms, thereby leading to sex-specific traits manifested in behavior, physiology, and morphology. Male and female generally have different alleles or even different genes, responsible for the regulation of their sexual morphology [41]. A total of 47 sex-related gene families were identified from the M nipponense androgenic gland transcriptome based on the functional annotation of non-redundant transcripts (Table 2). Most of these functional genes were identified based on comparisons with published data for other species [9]–[11], [42]–[44], but some were identified according to their GO classification.

**Table 2:** Sex- or reproduction- related ESTs identified in the androgenic gland transcriptome of *M. nipponense.*

Transcripts	Length (bp)	E-value	Accession number	Hits
Insulin-like androgenic gland specificfactor (IAG)	854–2541	0	ref|NP_563742.1|	4
DEAD-box ATP-dependent RNA helicase	397–4215	0	ref|NP_187299.1|	42
Sex-lethal	1981–2058	0	ref|XP_003250344.1|	2
Transformer-2 protein	452–4421	0–1.00E-05	ref|XP_001513669.2|	8
Extra sex combs	1291–1534	0	gb|AAC05332.1|	2
FTZ-F1	382–2037	0	gb|ADK46871.1|	5
FOXL2	3196	8.00E-42	gb|ABP63571.1|	1
ECM	437–5934	0	ref|NM_179755.3|	5
FEM1	2066–2741	0	ref|NP_001153369.1|	4
DHH	377–2647	0–3.00E-28	ref|NP_193244.2|	13
START	430–2598	0–2.00E-16	ref|NM_124797.4|	8
GATA	382–2598	0–7.00E-14	ref|NM_001036712.1|	31
Pumilio	363–2504	0–6.00E-07	ref|NM_001202701.1|	9
Argonaute	380–3427	0–2.00E-39	ref|NM_179453.2|	13
Chromobox protein	2954–3086	0	gb|BT060302.1|	2
Akt	1520–2755	0	ref|NM_128222.5|	3
Wnt	854–2802	0–4.00E-40	gb|EFX66479.1|	8
Heat Shock Protein	275–2541	0–9.00E-33	gb|AAM67147.1	86
Male reproductive-related protein	691–6177	0–8.00E-42	gb| EF364539.1|	12
DNM	638–2896	4.00E-20	gb|EF208559.1|	8
Tsunagi	798	0	gb|EFX81381.1|	1
Gustavus	3301	0	gb|GU462157.1|	1
Cytochrome P450	352–2369	0–9.00E-14	ref|NP_189262.1|	175
Cathepsin A	2384	0	gb|ADO65982.1|	1
Cathepsin B	370–1470	0	gb|EFA01289.1|	5
Cathepsin D	731–923	0	sp|Q05744|	2
Cathepsin L	1406–1717	0	emb|X99730.1|	2
PIGS	443–3186	0	ref|NP_187374.2|	4
Cyclin	125–4125	0–7.00E-43	ref|NP_180363.1|	93
CDC	456–287	0–9.00E-41	ref|NP_566911.1|	24
BCS	364–1566	0–1.00E-12	emb|CAB52469.1|	7
Cyclophilin	728–1819	0–1.00E-05	ref|NP_194968.2|	15
WWP	3123–4408	0	ref|NP_197706.1|	2
Dynactin	796–1677	0	gb|ACD13590.1|	3
Flotillin	370–5757	0–7.00E-30	ref|NP_197908.1|	9
PSMB	871–1721	0	ref|NP_565156.1|	15
TCP	363–2504	0–7.00E-33	ref|NP_172520.1|	25
Ubiquitin-conjugating enzyme E2	365–4010	0–6.00E-44	ref|NP_565440.1|	67
E3 ubiquitin-protein ligase	352–10699	0–8.00E-29	ref|NP_192209.2|	177
Ubiquitin carboxyl-terminal esterase L3	1204	0	gb|ACO36738.1|	1
Ubiquitin carboxyl-terminal hydrolaseisozyme L5	1757	0	gb|ACM43511.1|	1
Ubiquitin carboxyl-terminal hydrolase	354–3818	0–4.00E-39	ref|NP_563719.1|	79
Ubiquitin-binding protein	1349	0	ref|XM_001180530.1|	1
Ferritin	172–1116	0–3.00E-22	gb|EU371046.1|	4
Ferritin heavy-chain subunit	524–1100	0	ref|NP_990417.1|	3
Ferritin light-chain subunit	4589	0	gb|FJ446525.1|	1
Ferritin peptide	972	2.00E-42	gb|DQ205422.1|	1

In the *M. nipponense* androgenic gland transcriptome generated in this study, an important series of transcription factors homologous known as sex-determination related genes in other species, were found through high-throughput technology, these included insulin-like androgenic gland specific factor (*IAG*), transformer-2 (*tra-2*), sex lethal (*sxl*), and so on. *IAG* function is believed to be similar to that of the isopod AG hormone, which was the first to be structurally elucidated and belongs to the insulin superfamily of proteins, considered as key regulator of male sex-determination [23]. Recently, its homologs were found to be expressed in the AGs of several decapod crustaceans. The gene has been cloned and studied in several crustacean species, including *Callinectes sapidus*, *M. rosenbergii*, *Macrobrachium nipponense,* and *Penaeus monodon* [22]–[27]. IAG was exclusively and abundantly expressed in the androgenic gland in *M. nipponense* based on RT-PCR analysis [24]. Further studies will focus on the precise function of this gene in *M. nipponense* and determine how this gene will affect male sex-determination in this species. *Sxl* and *Tra-2* have been cloned from *M. nipponense*, based on the construction of a testis cDNA library. During embryogenesis, *Sxl* and *Tra-2* reached their highest levels at the *nauplius stage.* During the larval stage, *Sxl* and *Tra-2* have similar expression patterns, in which the expression of both genes gradually increased from day 1 post hatching (L1) to day 10 (L10) and decreased to their lowest levels at the end of metamorphosis, suggesting that both *Sxl* and *Tra-2* are involved in *M. nipponense* sex-determination. A reasonable explanation is that *Sxl* may act with *Tra-2* to play complex and important roles in embryogenesis, metamorphosis, somatic sexual development, and sex-differentiation [45], [46].

FTZ-F1 is a member of the nuclear hormone receptor superfamily [47], [48] and it was originally considered to be involved in the regulation of the transcription of *fushi tarazu* (*ftz*) in *Drosophila* [49]. Two isoforms, α- and β-FTZ-F1, are transcribed from the same gene. Α-FTZ-F1 expressed in the early-stage of embryo, containing *ftz* expression, whereas β-FTZ-F1 expressed in the late stage embryo when *ftz* is absent [50]–[51]. Its homologues, an essential factor in sex determination in mammals, have been identified in human, mouse and a number of teleost species [52]–[55]. FEM1 is a signal-transducing regulator in the *C. elegans* sex-determination pathway, played an essential role in sex determination in *C. elegans* [56]. The homologues of FEM1, including FEM1A, FEM1B and FEM1C, have been identified in human and house mouse. The expression of a single FEM1 transcript and protein showed no significant difference in both sexes, suggesting its activity was regulated by primarily posttranscriptional and posttranslational [56]. The genes, introduced above, were also reported in previous study as important sex-determination genes [11], indicating these genes are valuable sex-determination genes for further studies.

Chromobox proteins, members of a conserved family, are thought to be located on the W chromosome in chicken [57]. Female heterogamy (ZW) exists in both *M. nipponense* and *P. monodon* since this protein was also identified in the ovary cDNA library of these species [9]–[10], [58]; it is involved in the packaging of chromosomal domains into representative heterochromatic states [59]. It has been speculated that the sex of *M. rosenbergii* is determined by both heterogamous (ZW) females and homogamous (ZZ) males [60]. However, chromobox proteins existed in both the testis and the androgenic gland, implying female heterogamy is the main factor in *M. nipponense* sex-determination.

Besides, several important sex-differentiation related genes were also identified in the current study. Many studies have shown that ubiquitin may be relevant to heat shock proteins [61]–[63]. For instance, heat shock proteins (HSP) may help ubiquitin and target non-repairable proteins to the proteasome [64]. In the present study, a total of 6 ubiquitin gene families and 8 HSP gene families were found in the transcriptome. The ubiquitin–proteasome affects many biological processes, including cell degradation and protein homeostasis maintenance. A series of ubiquitin-conjugating enzyme E2 (E2) transcripts are essential for oogenesis and spermatogenesis, as its expression levels are significantly different at the various stages of testis and ovary development [65]. Ubc9, which belongs to the family of ubiquitin-conjugating enzyme E2, has been confirmed to have effects on *M. nipponense* embryogenesis and oogenesis [66]. A series of E3 ubiquitin-protein ligase (E3) transcripts are involved in the regulation of binding the target protein substrate and transferring ubiquitin from the E2 cysteine to a lysine residue on the target protein [67]. Ubiquitin carboxyl-terminal hydrolase L3 (*uch-l3*) and ubiquitin carboxyl-terminal hydrolase L5 (*uch-l5*) were also found in this transcriptome, functioning during the meiotic differentiation of spermatocytes into spermatids [68]. Uchl3 and uchl5 are widely expressed throughout the entire body, including the testis/ovary, neuronal cells, and in spermatocytes and spermatids. HSPs are a class of functionally related proteins found in all living organisms, including bacteria, plants, animals, and humans [69]. Their expression increases under stressful conditions, such as exposure to elevated temperature. Heat shock proteins have several different families classified according to their molecular weight [70], [71]. For example, Hsp 90 prevents protein aggregation and increases Hsp expression [72]. Hsp90 is involved in *M. nipponense* ovarian development, playing a regulatory role in ovary maturation [73]. Heat shock cognate 70 kDa proteins are synthesized in haploid cells during spermatogenesis and are mainly activated at the spermatid stage [74]. The ubiquitin and HSP genes in this study may work in combination, affecting the sex-determination process.

Cathepsins, which are contained in many types of cells in all animals, tear apart other proteins. In crustaceans, cathepsin A has functions in the innate immune system [75]. Cathepsin B was reported to control the developmental processes in insects and other arthropods. Cathepsin D is necessary for the formation of the yolk [76]. Cathepsin L regulates the development of the ovary in many species, including *L. vannamei*, *M. nsis*, and *Bombyx* sp. Cathepsin A, B, D, and L were found in this study, as the same as the *M. nipponense* testis cDNA library, implying these four types of cathepsins may play an essential role in the male developmental process. In particular, cathepsin A and D may be especially important because these two cathepsins were not found in the *M. nipponense* ovary cDNA library.

Cyclins are necessary for the progression of cells through the cell cycle by activating the cyclin-dependent kinase enzymes [77]. In this study, a dozen transcripts, related to the cyclin family subtype were found. These may play essential roles in the reproductive and developmental processes, they included cyclin A, B, D, and cyclin-dependent kinases (CDKs). Cyclin A and D are involved in many mechanisms, including egg maturation, meiosis, and the normal cell cycle [78], [79]. Cyclin B is involved in mitosis; it rises abruptly through the cell cycle until mitosis and then decreases significantly because of the degradation of cyclin B [80]. CDKs, present in all eukaryotes, are also involved in regulating transcription, mRNA processing, and the differentiation of nerve cells and their regulatory function in the cell cycle and are considered to be evolutionarily conserved [81].

### Novel Genes from Androgenic Gland

In this study, we compared the expression levels of genes in the *M. nipponense* androgenic gland with genes in the transcriptomes of other sex glands, including the vasa deferentia, ovaries and testes. These transcriptomes were constructed by our lab and the literatures being written currently. We divided the androgenic gland transcriptome genes into two main parts. One comprises the genes specifically expressed in the androgenic gland (21,246 genes) (**Table S6**), and the other comprises the genes generally expressed in sex glands (35,400 genes) (**Table S7**). A total of 40 candidate novel genes were found in the androgenic gland. Expression levels of these genes were significantly higher in the androgenic gland compared to the other sex glands, suggesting that they potentially play important roles in the sex-determination mechanism of *M. nipponense*. In the group of genes specifically expressed in the androgenic gland, 24 were considered to be the candidate genes because their expression levels were >1,000 (**Table 3**). In the generally expressed gene group, the *IAG* expression pattern was used as reference because it plays an essential role in the androgenic gland. A total of 16 genes were found to have similar expression patterns to *IAG* (**Table 4**). Interestingly, of the 40 candidate genes, 10 were uncharacterized proteins, the functions still remain unclear, but they may play essential roles in the androgenic gland because they are highly expressed there. However, this has yet to be confirmed.

**Table 3:** High expression levels of genes in androgenic gland specially expressed gene group.

	AG
Slow-tonic S2 tropomyosin	35859.99
Slow-tonic S2 tropomyosin	35322.07
Slow tropomyosin isoform	6497.87
Beta-glucosidase 23	5577
Slow tropomyosin isoform	4756.74
Transketolase	4295
Nitric oxide synthase	3159.14
Uncharacterized protein	2652.28
Conserved hypothetical protein	2213.42
Calmin-like protein	1896.84
Similar to ankyrin 2,3/unc44	1866.91
Glycogen debranching enzyme-like	1855.11
Dihydropteroate synthase	1838
Glutamine synthetase cytosolic isozyme 1–2	1655.61
Aquaporin PIP2-1	1384.64
Endoplasmin-like protein	1352
Hypothetical protein CaO19.7238	1231.24
Glycine cleavage system H protein	1171
Tubulin binding cofactor C domain-containing protein	1163
Uncharacterized protein	1141
Uncharacterized protein	1041
Uncharacterized protein	1023.71
ATP synthase subunit gamma	1015
40S ribosomal protein S6-1	1012

Note: AG means androgenic gland. Genes in this table only expressed in androgenic gland and were not detected in vasa deferentia, ovary and testis. Nmuber means the gene expression level in androgenic gland.

**Table 4:** Genes with similar expression pattern with IAG in generally expressed gene group.

	AG	VD	O	T
Troponin I	64234.01	1301.32	9	97.62
Uncharacterized protein	60606.33	3145.35	4.07	123.7
Uncharacterized protein	50406	3535.28	11	60.13
Uncharacterized protein	35327.41	2353.87	3.93	72.92
Troponin T-like isoform 4	34579.06	202.28	33.54	35.08
SERCA	21300.36	261.57	9.35	63.52
**IAG**	**20198.69**	**4912.33**	**6**	**5**
Lit v 3 allergen myosin light chain	20123	946.23	1	29
Actin 1	19808.65	138.52	3	3
Chlorophyll a–b binding protein 4	16184	32	14	15
Troponin C2	15186.76	1136.6	1	36.2
Beta-adaptin-like protein C	14389	24	22	18
Chlorophyll a–b bindingprotein CP26	14270	10	11	7
Sarcoplasmic calcium-binding protein	13419	37	7	10
Uncharacterized protein	13217	597	24	22
Muscle LIM protein isoform 1	11554.41	1946.92	6.67	18

Note: AG means androgenic gland. VD indicated vasa deferentia. O indicates ovary. T means testis. Nmuber means the gene expression level in each tissue.

Tropomyosin (4 genes) and troponin (3 genes) may have important effects on the androgenic gland (**Table 3** **,** **Table 4**). Tropomyosin is highly expressed in the androgenic gland, implying it plays a vital role there. Tropomyosins control the function of actin filaments in both muscle and non-muscle cells. They are often divided into muscle and non-muscle tropomyosin isoforms. In the muscle sarcomere, muscle tropomyosin isoforms regulate interactions between actin and myosin, playing a pivotal role in regulated muscle contraction. Non-muscle tropomyosin isoforms function in all cells, controlling and regulating the cell’s cytoskeleton and other key cellular functions [82]. Troponin is a complex of three regulatory proteins (troponin C, troponin I, and troponin T), which are integral to contraction in skeletal and cardiac muscles [83]. Troponin is attached to the protein tropomyosin and lies within the groove between actin filaments in the muscle tissue [83]. The high expression levels of both tropomyosin and troponin families imply that genes from these families act together affecting the androgenic gland. Furthermore, many genes from these 40 candidate novel genes were involved in glucose metabolism, including beta-glucosidase 23, glycogen debranching enzyme-like, and glutamine synthetase cytosolic isozyme 1–2. Research on these potential candidate novel genes and analysis of the relationship between them and genes expressed in other tissues, including identifying the genes up- and downstream of sex-determination related genes, may significantly advance all-male aquaculture having positive economic effects.

### Identification of Molecular Markers

**Figure 4:**
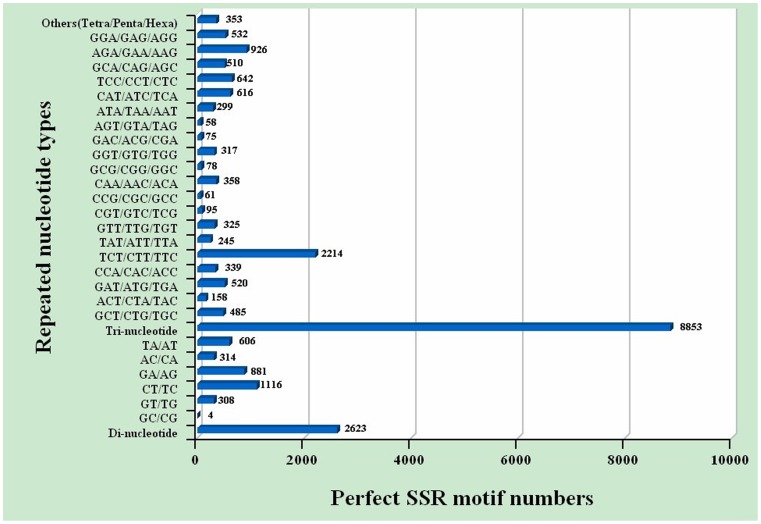
Distribution of simple sequence repeat (SSR) nucleotide classes among different nucleotide types found in the transcriptome of *M. nipponense*.

In this study, a large number of SSR and SNP markers were obtained (**Table S8, Table S9**). A total of 12,437 SSRs were obtained in the transcriptomic dataset, including 71.18% tri-nucleotide, 25.96% di-nucleotide, and 2.85% tetra/penta/hexa-nucleotide repeats (**Figure 4**). Among the tri-nucleotide repeat motifs, (TCT/CTT/TTC)n with a total of 2,214 SSRs and a frequency of 17.80% was the most common type, dramatically more than the other types of tri-nucleotide repeat motifs (**Figure 4**). There was a bias towards tri-nucleotide repeat motifs composed of C and T. (CT/TC)n, (GA/AG)n, and (TA/AT)n were the three dominant di-nucleotide types. Compared with the three previously constructed *M. nipponense* cDNA libraries, the volume in our study was a lot higher. In addition, 968 out of 12,437 EST-SSRs from the androgenic gland transcriptome were screened out. The frequency of these EST-SSRs was 11.4%. 673 SSRs were dinucleotide repeats, accounting for 69.5% of all SSR sequences, followed by 239 trinucleotide repeat SSRs and 26 tetranucleotide repeat SSRs. The SSRs with 5 or more nucleotides accounted for 0.03%. One hundred thirty-five pairs of primers were synthesized randomly, and 72 pairs of these exhibited clear bands. Finally, 14 markers were polymorphic in the test population of 32 individuals. The repeat motifs are listed in Table 5 and the repetitions ranged from 5 to 26. The average allele number was 7 per locus, ranging from 4 to 13. The observed heterozygosity ranged from 0.4125 to 0.8938 and expected heterozygosity ranged from 0.6786 to 0.9332. The PIC value ranged from 0.613 to 0.899. Four loci (E-WXM9, E-WXM10, E-WXM11, and E-WXM62) showed significant departure from HWE in the test population (*P*<0.05) (**Table 5**). These 14 EST-SSR markers may to some extent improve further studies, including those on the construction of linkage groups. We intend to isolate more SSR markers from this transcriptome in the future.

**Table 5:** Characterization of 14 polymorphic EST-SSR makers in *M. nipponense.*

Locus	Primer sequence(5-3)	Size(bp)	*T*a(°C)	*N*a	*H*o	*H*_E_	PIC
E-WXM3	F:AGTTGCTGTGCCACCTGC	213–278	58	5	0.7135	0.8235	0.802
	R:AAGCCACCACTGCCCTGT						
E-WXM7	F: GCAGTTTACATATCGGAACA	295–336	54	8	0.6625	0.8388	0.821
	R: CGATCTGGTGGAGTTGAT						
E-WXM9	F:AACATTAAACCGTCTGAA	338–402	54	5	0.5000	0.8297	0.899+
	R:ACCCTATGCGTCCTAACT						
E-WXM10	F: ACCCATCCAATCAAACAC	241–254	54	4	0.5312	0.7178	0.791+
	R:TGAGCATCAGCAGCATTA						
E-WXM11	F:GTCCGAGCCTCCTTCTTC	234–248	56	4	0.4125	0.6786	0.613+
	R:TCCACCTCCTTTGCCACT						
E-WXM14	F:CCCTCGTGAGATGATGTG	348–416	55	7	0.6875	0.8333	0.799
	R:CAGGACTGAGTGGCAAAA						
E-WXM16	F:GCAGTGAATTATTGTGCTCCTA	303–335	56	7	0.6700	0.8145	0.776
	R:TCCTGTGGCTCTGCTTTG						
E-WXM24	F:AAGGTTCGTTCATGCGTTAG	289–358	56	6	0.7923	0.8520	0.725
	R:CGGATATTATTTCTGTTGGGTT						
E-WXM29	F: GATTTATCTCAGGTGGGT	182–194	54	4	0.7500	0.8436	0.874
	R: ACTAAGGAAACAGGCATT						
E-WXM33	F: AACATTCACTGGCTCTTCG	188–236	54	13	0.8938	0.9216	0.890
	R: CCACTACTGTTTCTATCCACC						
E-WXM62	F:GCTTGTAGAAACCCGTAG	134–189	52	13	0.7188	0.9132	0.672+
	R:CTCTGACCTGCTTAGAAAA						
E-WXM89	F:GTTACCCAACCAGGCATT	282–333	56	10	0.7688	0.8249	0.682
	R:GCATTTTCAGACGCACATAA						
E-WXM93	F:GCCAAGAAGCCGAAGACT	235–298	56	8	0.7138	0.8652	0.798
	R:TTTTGACAGCAAGGGGAT						
E-WXM147	F:ATTGTCGTAGGCTCACGT	243–280	50	9	0.8688	0.9332	0.657
	R:AAAATTGGTCTTGCTCCC						

Note: *T*a, annealing temperature; *N*a number of alleles; *H*_O_ observed heterozygosity; *H*_E_ expected heterozygosity; PIC, polymorphic information content.

+ indicates significant deviation from *HWE* (P<0.05).

**Figure 5:**
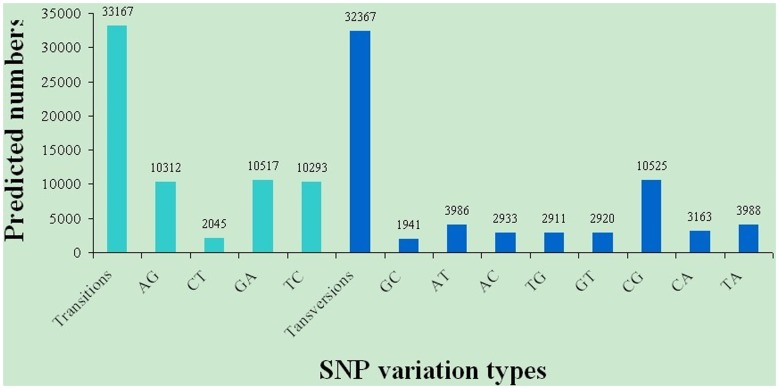
Distribution of putative single nucleotide polymorphisms (SNP) in the transcriptome of *M. nipponense*.

SNPs were identified from alignments of multiple sequences used for contig assembly. By excluding those that had a mutation frequency of bases less than 1%, a total of 65,535 SNPs were obtained, of these 33,167 were putative transitions (Ts) and 32,367 were putative transversions (Tv), giving a mean Ts: Tv ratio of 1.02∶1 across the *M. nipponense* androgenic gland transcriptome (**Figure 5**). The AG/GA, CT/TC, and CG/GC SNPs were the most common. In contrast, CA/AC, AT/TA, and TG/GT types were the fewest SNP types because of the differences in the base structure and the number of hydrogen bonds between different bases. Compared with the three existing *M. nipponense* cDNA libraries, the volume of SNPs in our study was also a lot higher. The transcriptomes, sequenced by Roche 454 GS FLX, generally have missing SNPs [11], [37], mainly because of the experimental methodology. However, there are no missing SNPs in this study, suggesting Illumina/Solexa sequencing is an ideal method for future transcriptome construction.

SSRs, or microsatellites, are polymorphic loci present in genomic DNA, consisting of repeated core sequences of 2–6 base pairs in length [84]. SNPs (single-nucleotide polymorphisms) are the most common type of variation in the genome. SNPs provide the best genome coverage for analyzing the performance and production of traits. Genome with high-density SNP coverage is a powerful tool for whole genome association studies because it allows for the detection of linkage disequilibrium [85]. Thus, based on the advantages of SSRs and SNPs, the development of such markers for this species was desirable. It is envisaged that the potential markers identified here within the ESTs will provide an invaluable resource for studying the evolution and molecular ecology of *M. nipponense*, for genome mapping, and quantitative trait loci (QTL) analysis. However, many of the putative *M. nipponense* SNPs identified could simply represent allelic variants and future studies are planned to validate which ones are real.

## Conclusion

This is the first report on the transcriptome of the *M. nipponense* androgenic gland by *de novo* assembly using the Illumina Hiseq2000. The 57,619 non-redundant transcripts identified and assembled will facilitate gene discovery in *M. nipponense*. A total of 47 sex-determination and sex-differentiation process related gene families were identified. Many candidate novel genes, potentially involved in the sex-determination mechanism, were identified in the androgenic gland for the first time and are worthy of further investigation. In addition, a large number of SNPs and SSRs were predicted and can be used for subsequent marker development, genetic linkage, and QTL analysis. Such findings generated by pyrosequencing in *M. nipponense* provide a new resource for future investigations in this economically important species, especially in understanding the sex-determination and differentiation mechanism of *M. nipponense*.

## Materials and Methods

### Ethics Statement

The prawns were obtained from the Tai Lake in Wuxi, China. We got the permission from the Tai Lake Fishery Management Council. *M. nipponense* is not an endangered or protected species in China, which can be used for experimental materials. All the experimental animal programs involved in this study were approved by committee of Freshwater Fisheries Research Institute, and followed the experimental basic principles. Androgenic gland from each prawn was sheared under MS222 anesthesia, and all efforts were made to minimize suffering.

### Prawn and Tissue Preparation

A total of 100 healthy adult male *M. nipponense* with a wet weight ranging from 4.9 to 6.2 g (average = 5.5 g), and a total length ranging from 6.1 cm to 7.2 cm (average = 6.8 cm), were obtained from Tai Lake in Wuxi, China (120°13′44″ E, 31°28′22″ N). These specimens were transferred to a 500 L tank and maintained in aerated freshwater at room temperature (26°C) for 72 h prior to tissue collection. The androgenic gland from 100 individuals was collected and immediately frozen in liquid nitrogen until used for RNA extraction for transcriptome sequencing, in order to prevent total RNA degradation.

### RNA Isolation for RNA-seq

The androgenic gland tissues from the 100 individuals were pooled to provide sufficient RNA for transcriptome sequencing. The androgenic glands were extracted under an Olympus SZX16 microscope. Total RNA was extracted by using the UNlQ-10 Column Trizol Total RNA Isolation Kit (Sangon) following the manufacturer’s protocol. The OD260/280 and OD260/230 should range from 1.8 to 2.0 and >2.0, respectively, to ensure the purity of the RNA sample. To guarantee the transcriptome quality, the total volume of the RNA sample was >5 µg. RNA integrity was confirmed using a 2100 Bioanalyzer (Agilent Technologies, Inc.) with a minimum RNA integrity number (RIN) value of 7.0. The samples for transcriptome analysis were prepared using a Truseq™ RNA Sample Prep Kit (Illumina) according to the manufacturer’s recommendations. Briefly, mRNA was isolated from >5 µg of total RNA using oligo (dT) magnetic beads. mRNA was cut into short fragments by adding fragmentation buffer. First-strand cDNA was synthesized using random hexamer-primers, taking these short fragments as templates. RNaseH, buffer, dNTPs, and DNA polymerase I was used to synthesize second-strand cDNA. Short fragments were purified with Takara’s PCR extraction kit (Takara Bio, Inc.). Sequencing adapters were ligated to short fragments and resolved by agarose gel electrophoresis. Proper fragments were selected and purified and subsequently PCR amplified to create the final cDNA library template.

### Analysis of the Transcriptome Results

The transcriptome was sequenced using the Illumina HiSeq™ 2000. Four fluorescently labeled nucleotides and a specialized polymerase were used to determine the clusters base by base in parallel. The size of the library was approximately 200 bp and both ends of the library were sequenced. The 200 bp raw paired-end reads were generated on the Illumina sequencing platform. Image deconvolution and quality value calculations were performed using Illumina GA pipeline v1.6. The raw reads were cleaned by removing adaptor sequences, empty reads, and low quality sequences (reads with unknown sequences ‘N’ or less than 25 bp). The clean reads were assembled into non-redundant transcripts using the Trinity, which has been developed specifically for the *de novo* assembly of transcriptomes using short reads. To obtain non-redundant transcripts, we removed short sequences (100 bp in length) and partially overlapping sequences. The resulting sequences were used for BLAST searches and annotation against the Nr protein, the Swissprot, the COG, and the KEGG databases using an E-value cut-off of 10^−5^. Functional annotation by GO terms (www.geneontology.org) was analyzed by the Blast2go software. The COG and KEGG pathway annotations were performed using Blastall software against the COG and KEGG databases.

### Expression Analysis

The abundance of each tag was normalized to one transcript per million, in order to allow comparison between various sex glands, including the androgenic gland, vasa deferentia, ovaries and testes. The raw reads were cleaned by removing low quality sequences including ambiguous nucleotides and adaptor sequences. The calculation of unigene expression was conducted by using RSEM software [86]–[87].RSEM is an accurate and user-friendly software tool for quantifying transcript abundances from RNA-Seq data. It is particularly useful for quantification with de novo transcriptome assemblies because it does not rely on the existence of a reference genome [86].

### Molecular Marker Detection

All ESTs from the *M. nipponense* androgenic gland transcriptome with a total number of 17,060 were converted into FASTA format, and screened for the presence of SSRs using SSRHunter software (http://www.bio-soft.net). The primers were designed using PRIMER 5.0 software (http://www.bbioo.com/download/58-166-1.html). Thirty-two wild oriental river prawn individuals were collected as a test population from Taihu Lake in China. Genomic DNA was extracted from the muscle of these individuals using traditional proteinase-K digestion and phenol–chloroform extraction protocols [88]. PCR amplification was carried out in a 25 µl reaction volume, containing 1×PCR buffer (Tiangen, Beijing, China), 30–50 ng genomic DNA, 0.25 µM for each primer, 150 µmol/l dNTPs, 1.5 mM MgCl_2_, and 0.5 U Taq DNA polymerase (Tiangen, Beijing, China). The PCR reaction conditions were as follows: the DNA was first denatured at 94°C for 3 min; followed by 30 cycles of denaturation at 94°C for 30 s, annealing at 50–58°C for 30 s, and elongation at 72°C for 40 s; with a final elongation at 72°C for 10 min. PCR products were size-fractionated on 6% polyacrylamide gels by silver staining. The pBR322 DNA/BsuRI (Hae III) marker (Fermentas, Shenzhen, China) was used to determine the allele sizes. POPGEN32 software was used to analyze allele frequencies, *Ho*, and *He* for each locus and statistical significance of *HWE* [89]. *PIC* was estimated using the formula of Botstein et al. [90].

#### Data deposition

The Illumina Hiseq 2000 reads of *M. nipponense* were submitted to www.ffrc.cn/gene/list.asp.

## Supporting Information

Table S1 **Summary of BLASTx results for contigs of** ***M. nipponense*** **.**
(XLS)

Table S2 **Summary of BLASTx results for contigs with ORF of** ***M. nipponense*** **.**
(XLS)

Table S3 **Categories of Gene Ontology of** ***M. nipponense*** **unique sequences.**
(XLS)

Table S4 **Categories of COG of** ***M. nipponense*** **unique sequences.**
(XLS)

Table S5 **KEGG summary of** ***M. nipponense*** **unique sequences.**
(XLS)

Table S6 **Summary of genes specifically expressed in androgenic gland.**
(XLS)

Table S7 **Summary of genes generally expressed in sexual gland.**
(XLS)

Table S8 **Summary of putative SSRs from** ***M. nipponense*** **transcriptome.**
(XLS)

Table S9 **Summary of putative SNPs from** ***M. nipponense*** **transcriptome.**
(XLS)
